# The Roles of Chromatin Accessibility in Regulating the *Candida albicans* White-Opaque Phenotypic Switch

**DOI:** 10.3390/jof7010037

**Published:** 2021-01-09

**Authors:** Mohammad N. Qasim, Ashley Valle Arevalo, Clarissa J. Nobile, Aaron D. Hernday

**Affiliations:** 1 Department of Molecular and Cell Biology, University of California-Merced, Merced, CA 95343, USA; mqasim2@ucmerced.edu (M.N.Q.); avallearevalo@ucmerced.edu (A.V.A.); cnobile@ucmerced.edu (C.J.N.); 2 Quantitative and Systems Biology Graduate Program, University of California-Merced, Merced, CA 95343, USA; 3 Health Sciences Research Institute, University of California-Merced, Merced, CA 95343, USA

**Keywords:** white-opaque switching, *Candida albicans*, chromatin, transcriptional regulation, heritability, cell fate decisions, histone modifying enzymes, chromatin remodeling enzymes, histone chaperone complexes, epigenetics

## Abstract

*Candida albicans*, a diploid polymorphic fungus, has evolved a unique heritable epigenetic program that enables reversible phenotypic switching between two cell types, referred to as “white” and “opaque”. These cell types are established and maintained by distinct transcriptional programs that lead to differences in metabolic preferences, mating competencies, cellular morphologies, responses to environmental signals, interactions with the host innate immune system, and expression of approximately 20% of genes in the genome. Transcription factors (defined as sequence specific DNA-binding proteins) that regulate the establishment and heritable maintenance of the white and opaque cell types have been a primary focus of investigation in the field; however, other factors that impact chromatin accessibility, such as histone modifying enzymes, chromatin remodelers, and histone chaperone complexes, also modulate the dynamics of the white-opaque switch and have been much less studied to date. Overall, the white-opaque switch represents an attractive and relatively “simple” model system for understanding the logic and regulatory mechanisms by which heritable cell fate decisions are determined in higher eukaryotes. Here we review recent discoveries on the roles of chromatin accessibility in regulating the *C. albicans* white-opaque phenotypic switch.

## 1. Introduction

Multicellular organisms are comprised of many phenotypically and functionally distinct cell types, the vast majority of which contain the same primary genomic sequence. How a single set of genomic “instructions” can reliably yield many distinct and heritable phenotypic states is a fundamental question in biology. We have begun to understand that a single genome can support many transcriptional programs, which in turn specify unique cell type specific patterns of gene expression, and ultimately establish distinct phenotypes. These cell types are often heritably maintained in an epigenetic manner following each cell division, and it has become increasingly apparent that chromatin structure and accessibility play important roles in the transcriptional regulation of cell type specificity.

*Candida albicans*, a unicellular polymorphic fungus, has evolved the ability to establish two transcriptional programs that give rise to two distinct cell types called “white” and “opaque” based on their appearance at the single colony level. The white and opaque cell types are heritably maintained in an epigenetic manner through thousands of cell divisions with no change to the primary sequence of the genome [1,2]. A growing body of literature has identified numerous similarities between the molecular mechanisms governing the *C. albicans* white-opaque switch and those that underlie heritable cell type differentiation in higher eukaryotes [3,4,5,6,7]. Since a similar heritable phenotypic switch is not observed in the classic model yeast *Saccharomyces cerevisiae*, *C. albicans* has emerged as a compelling “simple” and genetically tractable eukaryotic model system to study heritable transcriptional programs in higher eukaryotes.

The *C. albicans* white and opaque cell types are established and maintained by distinct transcriptional programs that lead to a wide range of phenotypic differences between the two cell types. These include differences in metabolic preferences, mating competencies, cellular morphologies, responses to environmental signals, interactions with the host innate immune system, and expression of ~20% of genes in the genome [3,4,8,9,10,11,12,13,14,15]. A variety of environmental cues have been identified that can bias the switch in favor of the white or opaque cell type. Growth in the presence of N-acetyl glucosamine, elevated CO_2_ levels, acidic pH, anaerobic conditions, genotoxic or oxidative stress, and 25 °C all promote white to opaque switching, while 37 °C in the presence of glucose triggers en masse opaque to white switching [1,2,16,17,18,19,20,21]. The destabilizing effect of elevated temperature on opaque cells is not universal, however, and opaque cells can be heritably maintained at 37 °C when grown on alternative (i.e., non-glucose) carbon sources [22]. Under standard switch permissive laboratory growth conditions (25 °C on Lee’s medium supplemented with 100 μg/mL uridine and 2% glucose, or other similarly comprised synthetic defined growth medium), phenotypic switching between the two cell types occurs stochastically at a frequency of approximately one switch event per 1000–10,000 cell divisions [16,17,18,19]. In other words, once established, each cell type is maintained through an epigenetic mechanism that is stably inherited over thousands of subsequent cell divisions.

**Figure 1:**
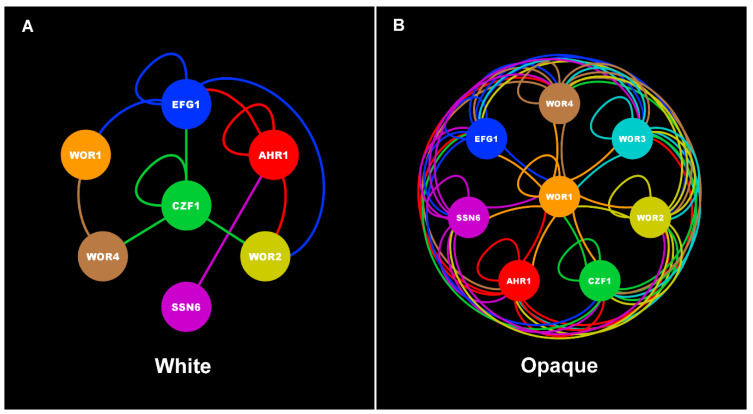
Core white and opaque transcriptional circuits. Colored lines indicate direct binding interactions between each TF (same color as their circular node) and their respective target genes within the white (**A**) and opaque (**B**) circuits. Data to create this figure was obtained from [4,9,22,23,25,27]. Figure was generated using Cytoscape [31].

The frequency of switching between the white and opaque cell types is controlled by a set of regulatory genes that encode seven sequence-specific DNA-binding proteins, i.e., transcription factors (TFs) (Wor1, Wor2, Wor3, Wor4, Ahr1, Czf1, Efg1), and one non-DNA-binding adapter protein (Ssn6). These eight switch regulators have been extensively characterized through genome-wide transcriptional profiling and chromatin immunoprecipitation (ChIP) experiments, as well as by genetic epistasis experiments [1,2,4,22,23,24,25,26]. Based on this work, these eight proteins have been shown to form the core of the transcriptional circuit that governs the establishment and heritable maintenance of the white and opaque cell types [4,9,22,23,25,27] (Figure 1). At the heart of this circuit lies Wor1, the “master regulator” of the opaque cell type, which is considered to be the key regulator involved in initiating the switch to, and heritable maintenance of, the opaque cell type [1,2,4,27]. *WOR1* expression, which is repressed in white cells and upregulated in opaque cells [1,2], triggers the formation of a highly intertwined regulatory network–consisting of the core regulators and all of their directly bound target genes–that is responsible for the establishment and heritable maintenance of the opaque cell type [1,2,3,4]. Remarkably, the high degree of interconnectivity in the core opaque transcriptional circuit (Figure 1) is similar to that observed in transcriptional circuits controlling stem cell maintenance and differentiation in mammals [3,28,29,30], suggesting that similar regulatory architectures may be common across eukaryotes to control analogous heritable transcriptional programs and their associated phenotypic outputs. Subsequent to the identification of the core transcriptional circuit controlling white-opaque switching, an additional 108 genes that encode known or predicted sequence-specific DNA-binding proteins have been identified as “auxiliary” regulators of the white-opaque switch [1,2,9,22] (Table 1). This auxiliary designation indicates that while these genes are known to influence the frequency of switching, the regulators that they encode have yet to be incorporated into the white-opaque transcriptional regulatory network through genome-wide chromatin association or other studies.

While much of the research on white-opaque switching to date has focused on regulatory TFs that bind directly to DNA in a sequence specific manner, an increasing body of literature has revealed important roles for factors that impact chromatin accessibility in regulating the dynamics of the switch [5,24,135,136,137,138,139,140]. Relatively little is known, however, at a mechanistic level, about how these factors influence switching. Since the ability of TFs to access their regulatory targets is substantially affected by chromatin landscapes [141], the roles that these chromatin accessibility factors play to influence white-opaque switching is an important avenue for future investigation.

Chromatin is composed of genomic DNA wrapped around four histone dimers, forming nucleosomes, as well as non-histone proteins that organize and stabilize chromatin structure. The regulation of chromatin is essential for cellular processes such as transcription, replication and repair, mitosis, and apoptosis [142]. The four histone dimers that make up the nucleosome core can be post-translationally modified either before or after their deposition into chromatin, adding an extra layer of information that is encoded on top of the primary sequence of the genome. These epigenetic modifications, which have unique context dependent functions, include acetylation, methylation, crotonylation, ubiquitinoylation, SUMOylation, and phosphorylation [143,144,145,146,147,148,149]. Although these modifications are considered epigenetic marks, most of them are not heritably transmitted from one cellular or organismal generation to the next. Histone modification is a reversible process that is mediated by specific enzymes that are classified as “writers”, such as histone lysine methyltransferases, that catalyze the addition of chemical modifications, and “erasers”, such as histone deacetylases, that remove chemical modifications [150]. Often, the writers and erasers function within multiprotein complexes that also contain proteins with specialized domains, such as bromodomains, that are classified as “readers”. Readers recognize specific histone modifications and regulate the specificity and enzymatic activity of their associated writers and erasers [151]. Additional factors that influence chromatin structure and accessibility include remodelers, which actively translocate or evict nucleosomes, and histone chaperones, which deposit histones into chromatin [152,153,154,155].

**Figure 2:**
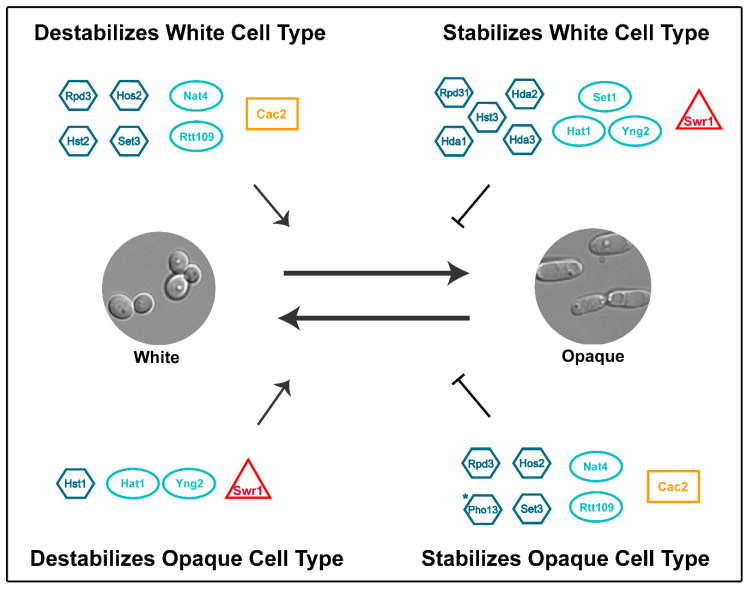
Roles of chromatin modifying enzymes in regulating the *C. albicans* white-opaque switch. White to opaque and opaque to white switching is indicated by the central black arrows. Smaller black arrows indicate proteins that act to promote switching in the white to opaque direction (upper left quadrant) or in the opaque to white direction (lower left quadrant), while black lines with crossbar indicate proteins that repress switching in the white to opaque direction (upper right quadrant) or in the opaque to white direction (lower right quadrant). Erasers are shown as blue hexagons, writers are shown as aqua ovals, chromatin remodelers are shown as red triangles, and histone chaperones are shown as orange rectangles. Note that Yng2 is a subunit of the NuA4 complex and that Cac2 is a subunit of the CAF-1 complex. *Pho13 has been shown to lack protein phosphatase activity and is instead involved in metabolism [159,160].

28 genes that encode known or predicted writers (eleven genes), erasers (fourteen genes), chromatin remodelers (one gene) and histone chaperones (two genes) have been analyzed for their roles in white-opaque switching [5,24,135,136,137,139,140,156] (Table 2). Eighteen of these genes are involved in white-opaque switching since their deletion significantly affected the frequency of white-opaque switching relative to an isogenic wildtype strain (Table 2). These genes encode proteins that fall into six functional categories with respect to their roles in white-opaque switching: (1) stabilizers of the white cell type that do not affect opaque cell stability (Set1, Rpd31, Hst3, Hda1, Hda2, and Hda3); (2) destabilizers of the white cell type that do not affect opaque cell stability (Hst2); (3) stabilizers of the opaque cell type that do not affect white cell stability (Pho13); (4) destabilizers of the opaque cell type that do not affect white cell stability (Hst1); (5) stabilizers of the white cell type that also decrease opaque cell stability (Hat1, Swr1 and Yng2); and (6) destabilizers of the white cell type that also increase opaque cell stability (Hos2, Set3, Nat4, Rtt109, Rpd3 and Cac2) (Figure 2). Below we review the current knowledge of the roles of these writers, erasers, chromatin remodelers, and histone chaperones in regulating the white-opaque switch.

## 2. Regulation of White-Opaque Switching by “Writers”

Five of the histone modifiers that influence white-opaque switching are “writers”, four of which are histone acetyltransferases (HATs) (Hat1, Rtt109, Nat4, Yng2) and one of which is a histone methyltransferase (HMT) (Set1). HATs modify histones by acetylating lysine residues at histone tails or at histone globular domains, while HMTs primarily modify histones by methylating lysine residues at histone tails. Each of the four HATs influence the stability of both the white and opaque cell types, with Hat1 and Yng2 playing opposing roles to Nat4 and Rtt109 (Figure 2) [5,24,140]. In contrast, the HMT Set1 specifically assists in the establishment of the opaque cell type by increasing the white to opaque switch frequency but does not affect opaque cell maintenance. In the following sections, we review the current knowledge of how these writers regulate the establishment and maintenance of the white and opaque phenotypic states.

### 2.1. Regulation of White-Opaque Switching by the NuA4 Histone Acetyltransferase Yng2

Histone acetyltransferases (HATs) are characterized by their substrate and cellular localization. Type A HATs modify nucleosomal histones and are localized in the nucleus, while type B HATs modify histones before they are deposited into nucleosomes and are localized in the cytoplasm [161]. Yng2, the catalytic subunit of the NuA4 HAT complex, is the only type A HAT known to regulate the white-opaque switch [5] (Table 2 and Figure 2). Although the mechanism by which NuA4 regulates the white-opaque switch is unknown, some mechanistic insights can be gleaned from the knowledge of how NuA4 regulates the yeast to hyphal cell transition [162]. The NuA4 complex is recruited to the upstream intergenic regions of hyphal-specific genes by Efg1 and, upon filament induction, an increase in H4 acetylation levels is observed at these sites [162]. This acetylation event is required for the recruitment of the SWI/SNF complex, which activates the hyphal-specific genes through its chromatin remodeling activity. Given that Efg1 is also a key white-opaque switch regulator and is bound upstream of many white- and opaque-specific genes, it is perhaps not surprising that genetic evidence suggests a similar process may be involved in regulating the white-opaque switch [162]. Notably, deletion of *YNG2* results in a nearly identical alteration of white-opaque switching as deletion of *EFG1* (~24-fold increase in white to opaque switching and ≥60-fold decrease in opaque to white switching) (Table 1 and Table 2). These findings suggest that H4K5 acetylation at white and opaque regulated genes that are directly bound by Efg1 likely plays an important role in stabilizing the white cell type and destabilizing the opaque cell type. Since NuA4 regulates the expression of hyphal-specific genes indirectly, through H4K5 acetylation-dependent recruitment of the SWI/SNF chromatin remodeling complex, it seems plausible that SWI/SNF complex-dependent chromatin remodeling may ultimately play a role in modulating the stability of the white and opaque cell types.

The NuA4 complex has also been implicated in the regulation of white-opaque switching through H2 and H4 acetylation-dependent recruitment of the SWR1 chromatin remodeling complex, which is responsible for depositing an H2A.Z histone variant into chromatin in exchange for a canonical H2A histone [163]. Deletion of *SWR1*, the major subunit of the SWR1 complex, results in an increase in white to opaque switching and a decrease in opaque to white switching, with similar fold changes in switch frequencies as observed for a *yng2* deletion mutant strain [5] (Table 2). The NuA4 HAT complex and the SWR1 chromatin remodeling complex share four subunits, and it was recently determined that the two complexes can merge to function as one unit depending on the morphological state of the *C. albicans* cell [164]. Taken together, these findings suggest that there is a complex interplay between histone modification and chromatin remodeling enzymes in regulating the stability of the white and opaque cell types. Studies in fungi and higher eukaryotes have shown that chromatin remodeling enzymes are often recruited to their target loci by a combination of factors, including histone modifications; however, the specific roles of these interactions in regulating cell type heritability is not understood in eukaryotes.

### 2.2. Regulation of White-Opaque Switching by the Histone Acetyltransferase Rtt109

Rtt109 is a type B HAT known to acetylate lysine 56 within the globular domain of histone 3 (H3K56) before the histone monomer is deposited into nucleosomes. This acetylation mark is especially important because the addition of a negative charge within the globular domains of histones reduces the electrostatic attraction between DNA and nucleosomes and thus destabilizes the affected nucleosomes [143]. This increase in DNA accessibility due to H3K56 acetylation influences the repackaging of chromatin after replication and DNA damage repair [165,166,167], and also plays an important role in anti-silencing and transcription at heterochromatic loci [168,169]. Interestingly, an increase in the levels of H3K56 acetylation is correlated with an increase in the rate of histone turnover at certain developmentally regulated genomic loci in higher eukaryotes [170]. This increase in the rate of histone turnover is, in turn, correlated with an increase in chromatin accessibility, and thus TFs (both activating and repressing) are more likely to bind to, and regulate the expression of, genes at these loci.

Rtt109 has been shown to play an important role in enabling the white to opaque transition and in the heritable maintenance of the opaque cell type [24]. In a *rtt109* deletion mutant strain, white cells were found to switch to the opaque cell type at a tenfold lower frequency than that of wildtype cells, and the resulting opaque cells were highly unstable [24]. In fact, opaque colonies of an *rtt109* deletion mutant strain were found to consist of a mixed population of both white and opaque cells, and only a subset of the elongated cells that resembled the opaque phenotype were found to express high levels of *WOR1* [24]. These findings suggest that the H3K56 acetylation mark plays a critical role in the stability of *WOR1* expression in opaque cells. Locus specific chromatin immunoprecipitation experiments by ChIP-qPCR revealed that H3K56 acetylation marks were differentially deposited at multiple loci upstream of genes that are differentially expressed between white and opaque cell types [22]. Indeed, H3K56 acetylation was found to be enriched within the upstream intergenic region of *WOR1* in opaque cells, relative to white cells [24]. How H3K56 acetylation is deposited in a cell type specific manner at the upstream intergenic regions of these differentially expressed genes remains an open question. While we do not yet know the specific mechanisms by which Rtt109 regulates the white-opaque switch, evidence suggests that Rtt109 regulates Wor1 accessibility to its own upstream intergenic region [24]. For example, we know that opaque cells expressing an ectopic copy of *WOR1* in an *rtt109* deletion mutant strain failed to heritably maintain the opaque cell type after the ectopic copy of *WOR1* was turned off [24], suggesting that Rtt109 activity and H3K56 acetylation enrichment within the *WOR1* upstream intergenic region are necessary for the stable maintenance of the *WOR1* positive feedback loop that is central to the heritability of opaque cells.

### 2.3. Regulation of White-Opaque Switching by the Histone Acetyltransferase Hat1

Hat1, a type B HAT that is part of the NuB4 complex, acetylates histone 4 (H4) tails at two different lysine residues (H4K5 and H4K12), and mediates the incorporation of free histones into nucleosomes [171]. The role of Hat1 in the NuB4 complex was confirmed by showing that reducing H4 levels mimicked inactivation of the NuB4 complex [140]. In *C. albicans*, a *hat1* deletion mutant strain displayed both increased white to opaque switching and decreased opaque to white switching [140]. In other words, the NuB4 complex seems to bias the switch towards the white cell type by both stabilizing the white cell type and destabilizing the opaque cell type. While the mechanism by which Hat1 influences the switch is unknown, we speculate that Hat1 may regulate the white-opaque switch by modulating H4 levels in chromatin, which would affect chromatin accessibility. In white cells, reduced H4 levels in a *hat1* deletion mutant strain could increase DNA accessibility for TFs binding to *WOR1* cis-regulatory elements, thus making it easier for Wor1 to initiate the autoregulatory positive feedback loop central to the white to opaque transition. In opaque cells, this increase in DNA accessibility could stabilize the *WOR1* positive feedback loop, thereby stabilizing the opaque state. Consistent with this idea, one would predict that white to opaque switching would be reduced in an *MTL* heterozygous *hat1* deletion mutant strain due to increased binding of the *MTL* **a**1/α2 heterodimer upstream of *WOR1*.

### 2.4. Regulation of White-Opaque Switching by the Histone Acetyltransferase Nat4

Deletion of *NAT4*, which encodes an N-terminal acetyltransferase (NAT), has been found to reduce white to opaque switching and increase opaque to white switching [135]. Thus, Nat4 tilts the scales in favor of opaque cell formation by destabilizing the white cell type and by stabilizing the opaque cell type. In *S. cerevisiae,* Nat4 acetylates the N-terminal serine residues of H4 and H2A [172]. While not much is known about the function of Nat4 in *C. albicans*, we briefly discuss a few unique properties of the *S. cerevisiae* Nat4 to highlight why NATs could be of interest to study in *C. albicans*.

Five NAT types have been identified in *S. cerevisiae* (NatA, NatB, NatC, NatD, NatE) [173], and with the exception of NatD, they all have human orthologs. Nat4, the catalytic subunit of NatD is the only NAT shown to regulate the white-opaque switch. Unlike the other NATs, NatD does not contain auxiliary subunits, and therefore does not require interacting partners for its enzymatic activity [174]. Interestingly, NatD recognizes a significantly longer N-terminal sequence for acetylation than the other NATs [174]. These unique properties of NatD suggest that this enzyme likely regulates the white-opaque switch by regulating the acetylation levels of H4 and H2A.

### 2.5. Regulation of White-Opaque Switching by the Histone Methyltransferase Set1

Set1 deposits methylation marks at lysine 4 of H3 (H3K4) via its SET domain-containing methyltransferase [175]. It is the only methyltransferase in *C. albicans* that modifies H3K4, and thus deletion of *SET1* in *C. albicans* results in a complete loss of H3K4 methylation [176]. In *S. cerevisiae* and higher eukaryotes, H3K4 methylation marks are hallmarks of transcriptionally active chromatin sites [175]. Studies in *S. cerevisiae* have shown that *SET1* is required for transcriptional silencing of the silent mating-type loci and telomeres [177]; however, the specific relationship between H3K4 methylation and transcription has not been investigated in *C. albicans*. A *C. albicans set1* deletion mutant strain has been shown to display increased white to opaque switching relative to the wildtype strain [135]. Unlike the HATs, which influence both white to opaque switching and opaque cell stability, the Set1 histone methyltransferase, however, does not affect opaque cell stability [135]. The mechanism by which Set1-dependent H3K4 methylation specifically influences white cell stability, without affecting opaque cell stability, is an intriguing area of interest for future studies.

## 3. Regulation of White-Opaque Switching by “Erasers”

Histone deacetylases remove histone acetylation marks and thus are often associated with a repressive function because of their roles in forming a condensed or “closed” chromatin state that restricts DNA accessibility [178]. Ten histone deacetylases (HDACs) (Rpd3, Rpd31, Hda1, Hda2, Hda3, Set3, Hos2, Hst1, Hst2, and Hst3) have been shown to influence white-opaque switching [24,135,137,158]. Three of these HDACs (Hda1, Hst3, and Rpd31) have no known roles in regulating the stability of the opaque cell type, and thus, similar to the Set1 HMT discussed above, appear to be white cell-specific modulators of the white-opaque switch [24,135,137]. These findings suggest that decreased chromatin accessibility, mediated by these HDACs either genome-wide or at specific regulatory loci, plays a role in maintaining cell type epigenetic heritability by modulating accessibility for TFs (activating and repressing). Below, we discuss the known roles of these HDACs in white-opaque switching.

Rpd3 is a histone deacetylase that acts on both H3 and H4 and has been shown to play a direct role in regulating *WOR1* expression in white cells [136]. Upon deletion of *RPD3*, H4 acetylation levels increase throughout the *WOR1* upstream intergenic region, and these elevated acetylation levels appear to directly influence white cell stability by increasing the accessibility of chromatin. Interestingly, the effect of *RPD3* deletion on white cell stability is dependent upon the mating type of the cell. In *MTL* heterozygous cells, where the *MTL***a**1/α2 heterodimer stabilizes the white cell type through direct repression of *WOR1* [14,27], deletion of *RPD3* results in an increase in *MTL***a**1/α2 binding upstream of *WOR1* and a decrease in *WOR1* expression [136]. Conversely, in an *MTL* homozygous (**a**/**a**) strain, where the *MTL***a**1/α2 heterodimer is not present, deletion of *RPD3* results in a decrease in white cell stability [136]. This decrease is presumably due to an increase in Wor1 accessibility to the *WOR1* upstream intergenic region, which would ultimately facilitate activation of the *WOR1* positive feedback loop that is central to the formation and stabilization of the opaque cell type. These results establish that white cell stability can be regulated through the modulation of interactions between regulatory TFs and their cis-regulatory target sites via histone deacetylation. Alternatively, HDACs could also remove histone acetylation marks from within open reading frames, thus changing their chromatin accessibility. Set3 and Hos2 were recently shown to form part of an HDAC complex in *C. albicans* that regulates expression of metabolic and morphogenesis related genes via this type of mechanism [179], and a similar mechanism has been proposed to explain how Rpd31 represses white to opaque switching [136]. While Set3, Hos2 and Rpd31 have been shown to regulate the white-opaque switch, their specific mechanisms of action on the switch have yet to be elucidated.

The SET3 complex, which includes the HDACs Set3, Hos2, and Hst1, has been shown to be intertwined at a genetic level with the transcriptional regulatory circuit that controls white-opaque switching. Genetic epistasis studies have highlighted an intriguing interaction between the SET3 complex and a key repressor of the white to opaque switch, Efg1 [135]. While deletion of *EFG1* alleviates Efg1-mediated repression of *WOR1*, and thus stimulates white to opaque switching and stabilizes the opaque state, deletion of either *SET3* or *HOS2* suppresses the *efg1* deletion phenotype and restores switching frequencies to near wildtype levels [135]. This suggests that the activation and sustained expression of *WOR1* that is central to opaque cell formation and stability is dependent not only on alleviation of Efg1 repression, but also on the activity of the SET3 complex. Since the SET3 complex has been shown to be a negative regulator of the protein kinase A (PKA) pathway [180], and Efg1 is believed to be a major regulatory target of the PKA pathway, [181,182,183] this suggests a potential mechanism for the genetic interactions observed between *SET3*, *HOS2*, and *EFG1*. Additional epistasis experiments revealed that deletion of *SET1*, encoding a methyltransferase, and the resulting loss of H3K4 histone methylation, suppresses the effect of *SET3* or *HOS2* deletion on white-opaque switching, revealing a complex interaction between these chromatin modifiers and the modulation of white and/or opaque cell stability. Although *SET3*, *HOS2* and *HST1* have all been shown to function as part of the SET3 complex, deletion studies indicate that Hst1 may act independently of the SET3 complex when regulating white-opaque switching [135,184]. Specifically, while Set3 and Hos2 both promote white to opaque switching and opaque cell stability, Hst1 appears to have no effect on white cell stability and acts to decrease opaque cell stability [135]. One potential explanation for this result could be that Hst1 may instead regulate opaque cell stability as a component of the SUM1-RFM1-HST1 complex, which has been shown to function as a repressor of sporulation-specific genes in *S. cerevisiae* [185] and a repressor of drug and oxidative stress resistance genes in *Candida glabrata* [186].

Similar to *SET3* and *HOS2* deletion phenotypes, deletion of *HST2* was found to result in a decrease in white to opaque switching, yet no alterations in opaque cell stability were detected [135]. This finding suggests that Hst2 is likely involved in destabilizing the white cell type. In contrast to *SET3* and *HOS2*, which are epistatic to *EFG1*, the *HST2* deletion phenotype is suppressed when *EFG1* is also deleted [29], suggesting that *HST2* may destabilize the white cell type by inhibiting or antagonizing *EFG1*. Hda1, which also plays roles in stabilizing the white cell type without influencing opaque cell stability, may also act through *EFG1*. *EFG1* expression is reduced in white cells when *HDA1* is deleted; however, the mechanism by which Hda1 influences *EFG1* expression has yet to be elucidated. Furthermore, *HDA1* expression is overall lower in opaque cells, relative to white cells [137], perhaps explaining why deletion of *HDA1* does not affect opaque cell stability. Hda1 has been proposed to form a complex along with Hda2 and Hda3 [158], potentially explaining the similarity in phenotypes between *HDA1*, *HDA2*, and *HDA3* deletion strains. In addition, deletion of any one of these three genes has been reported to result in at least a fivefold increase in *WOR1* expression [158], possibly due to a reduction in Efg1-dependent repression of *WOR1* transcription. Although Hda1 has been shown to promote sustained hyphal development via deacetylation of Yng2, leading to eviction of the NuA4 HAT complex from hyphal promoters [51,187], it is unclear whether a similar mechanism may be involved in white-opaque switching, as both Hda1 and Yng2 contribute to stabilizing the opaque cell type. Hst3 is a sirtuin class HDAC that removes H3K56 acetylation and stabilizes the white cell type [24], presumably by counteracting the white cell destabilizing effects of Rtt109, which facilitates sustained *WOR1* expression though increased H3K56 acetylation. It is interesting to note that Hst3 levels are reduced in response to genotoxic stress [24], providing a possible explanation for the up to eightfold increase in white to opaque switching observed when white cells are cultured in the presence of the cytotoxic drugs methyl methanesulfonate or hydroxyurea [24]. Understanding how these HDACs regulate the expression of key white-opaque transcriptional regulators, such as *WOR1* and *EFG1* to ultimately influence the relative stabilities of white and opaque cell types, is an important area of focus for future studies.

## 4. Potential Roles of “Readers” in Regulating White-Opaque Switching

“Reader” enzymes recognize or “read” post-translationally modified histone residues and possess a histone binding module, such as a bromodomain [188,189], that recognize specific histone residues with modifications. Bromodomain modules, for example, recognize histone lysine residues with acetylation marks [190]. While the roles of readers in regulating white-opaque switching have yet to be investigated, readers are involved in other important processes in *C. albicans*. For example, one reader of the lysine acetylation mark (Bdf1) and two readers of the crotonylation and acetylation marks (Taf14 and Yaf9), have been shown to play important roles in *C. albicans* pathogenicity [191,192]. NuA4-dependent acetylation upstream of hyphal-specific genes has been shown to result in the recruitment of the SWI/SNF chromatin remodeling complex via its bromodomain during hyphal induction [162]. In other fungi and higher eukaryotes, many readers have been identified and studied [193,194,195,196], but their functions have yet to be investigated in *C. albicans*. Several histone modifying enzymes and chromatin remodeling enzymes, which affect the white-opaque switch, also affect cellular differentiation and heritability in higher eukaryotes [197,198,199,200,201]. Readers are often components within histone modifying enzyme complexes and chromatin remodeling complexes, and thus, assist these large complexes in finding their target loci within the genome [202]. Therefore, it seems likely that readers are involved in regulating the *C. albicans* white-opaque switch, and this is an important unexplored area of interest for future studies.

## 5. Regulation of White-Opaque Switching by Chromatin Remodeling Complexes

Chromatin remodeling enzyme complexes modulate chromatin accessibility through the function of their ATPase-translocase domains. We can classify chromatin remodeling enzymes into four subfamilies, each of which carries out specialized functions [154]. ISWI and CHD complex subfamilies preferentially reduce chromatin accessibility by regulating the assembly and organization of nucleosomes. The SWI/SNF complex subfamily remodels chromatin by sliding or evicting nucleosomes, which generally increases chromatin accessibility [154]. The INO80 complex subfamily modulates chromatin accessibility by replacing canonical histones with histone variants, specifically targeting nucleosomes that flank transcription start sites. The SWR1 complex, a member of the Ino80 subfamily, is the only known regulator of this class that regulates white-opaque switching in *C. albicans* [5] and is discussed in more detail below.

### Regulation of White-Opaque Switching by the SWR1 Chromatin Remodeling Complex

*SWR1* encodes a chromatin remodeling enzyme that is responsible for the deposition of the histone variant H2AZ. The SWR1 complex, which is an ortholog of the human SRCAP complex, is a multiprotein complex responsible for replacing canonical histone H2A-H2B dimers with the histone variant H2A.Z-H2B dimers without disassembling the H3/H4 tetramer from DNA [163,203]. H2A.Z is a highly conserved variant of H2A that is found throughout all eukaryotes [204]. Developmentally regulated genomic loci show increased enrichment of H2A.Z relative to non-developmentally regulated loci [205]. H2A.Z is deposited specifically into the two nucleosomes that flank transcription start sites [206], and is essential in several higher eukaryotic organisms, but not in fungi [207,208]. In *C. albicans*, H2A.Z is enriched in white cells, relative to opaque cells, within the upstream intergenic region of *WOR1* [5]. The complex responsible for depositing this histone variant appears to play a role in stabilizing the white cell type and destabilizing the opaque cell type, as deletion of *SWR1* causes a significant increase in the white to opaque switch frequency and in the heritable maintenance of opaque cells [5]. Since H2A.Z variant enriched sites have been shown to correlate with slightly increased chromatin accessibility relative to canonical histones [209], it is conceivable that higher levels of H2A.Z inhibit expression of *WOR1* by facilitating the binding of a repressor protein within the upstream intergenic region of *WOR1*.

A similar phenotype is observed upon disruption of the NuA4 complex, which is known to recruit and/or promote chromatin-related enzymatic activities of the SWR1 complex [162,210]. Therefore, it is likely that NuA4 regulates the white-opaque switch by modulating the recruitment or enzymatic activity of Swr1, which in turn results in decreased H2A.Z deposition throughout the genome. The nucleosome editing function of the SWR1 complex is also controlled through H3K56 acetylation, which is catalyzed by Rtt109. High levels of H3K56 acetylation led to decreased levels of H2A.Z deposition genome-wide [211]. This is notable as H3K56 acetylation itself has been implicated in altering histone turnover rates [212], which consequently alters genome-wide chromatin accessibility. It remains an open question whether H3K56 acetylation regulates the white-opaque switch by modulating the enzymatic activity of the SWR1 complex, or whether H3K56 acetylation directly regulates the white-opaque switch by modulating histone turnover rates.

## 6. Regulation of White-Opaque Switching by Histone Chaperone Complexes

The highly basic amino acid composition of histones makes them predisposed to aggregation and promiscuous histone-DNA interactions, thus necessitating a diverse network of histone chaperones to orchestrate the assembly and integration of histones into chromatin [152,153,213]. Below, we focus our discussion on the evolutionarily conserved histone chaperone complexes HIR (HIRA in humans) and CAF-1, and their roles in regulating the white-opaque switch in *C. albicans*. CAF-1 primarily assembles nucleosomes in a replication dependent manner [214,215], whereas HIR functions independent of replication [216,217]. Importantly, the replication coupled nucleosome assembly function of CAF-1 is conserved in humans [214,215]. Both chaperone complexes are essential in higher eukaryotes [218,219], which has complicated efforts to investigate their functions in cell type formation and maintenance. The *C. albicans* white-opaque switch provides a unique and robust alternative system to investigate the functions of these highly conserved chaperone complexes in higher eukaryotes.

Studies in both *S. cerevisiae* and human HeLa cells have revealed that the HIR and CAF-1 complexes modulate nucleosome dynamics [220], which in turn affect chromatin accessibility. Other than their replication dependent functions, these two enzymes have also been shown to have several overlapping functions that are unrelated to replication. Recent work in *C. albicans* has shown that they function similarly to their orthologs in *S. cerevisiae*. Deletion of *C. albicans HIR1,* a subunit of the HIR complex, had no effect on white-opaque switching, while deletion of CAC2, a subunit of CAF-1 complex, resulted in an overall increase in switching in both directions [139]. On the other hand, deletion of a subunit of both chaperone complexes in *C. albicans* has been shown to lead to reduced opaque cell stability, as evidenced by wildtype levels of white to opaque switching and a sixfold increase in opaque to white switching [139]. These results alone do not definitively point to a specific chaperone complex responsible for regulating opaque cell stability; however, they do reveal that nucleosome dynamics can significantly affect cell type maintenance in the context of the white-opaque switch. Modulating nucleosome dynamics has a significant effect on chromatin accessibility [141], and recent studies have acknowledged the impact of chromatin accessibility on cell type specification and maintenance [170,200,221,222,223]. It is possible that opaque cells, more so than white cells, depend on increased chromatin accessibility to maintain their cell type specific transcriptional program, which could explain why deleting subunits of the HIR and CAF-1 complexes have dramatic effects on opaque cell stability.

## 7. Conclusions

Most chromatin research in *C. albicans* has focused on the roles of chromatin in regulating cellular processes such as transcription, replication, repair, mitosis, and apoptosis [35]. Recently, this focus has shifted to investigating how chromatin and chromatin modifiers regulate cell type specification and heritability. This avenue of research has led to significant insights into how chromatin regulates these fundamental biological processes. Many of these insights come from studies in higher eukaryotes; however, the inherent complexity of myriad possible cell type lineages and large genome sizes has slowed progress in the field.

**Figure 3:**
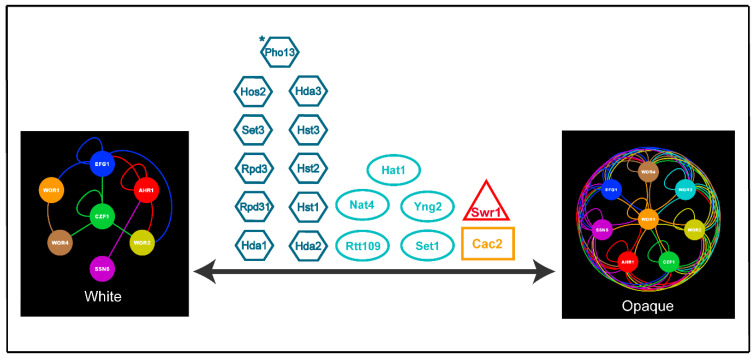
Summary illustration of the roles of chromatin regulating proteins in modulating the *C. albicans* white-opaque switch. Colored lines within the core white and opaque transcriptional circuits indicate direct binding interactions between each TF (same color as their circular node) and their respective target genes. Data to create the transcriptional circuits was obtained from [4,9,22,23,25,27]. Transcriptional circuits were generated using Cytoscape [31]. White to opaque and opaque to white switching is indicated by the central black arrows. Erasers are shown as blue hexagons, writers are shown as aqua ovals, chromatin remodelers are shown as red triangles, and histone chaperones are shown as orange rectangles. Note that Yng2 is a subunit of the NuA4 complex and that Cac2 is a subunit of the CAF-1 complex. *Pho13 has been shown to lack protein phosphatase activity and is instead involved in metabolism [159,160].

The white-opaque switch in *C. albicans* is not hindered by the same challenges as higher eukaryotes, and thus represents an attractive alternative model system for investigating the mechanisms by which chromatin dynamics regulate cell type specification and heritability. We have reviewed the roles of chromatin regulating proteins in modulating the white-opaque switch and the heritability of white and opaque cell types in *C. albicans* (summarized in Figure 3). Most of these proteins have been shown to also affect cellular differentiation and heritability in higher eukaryotes, thus supporting the overall relevance of this research. Future studies on chromatin regulating proteins in *C. albicans* will certainly lead to significant insights into the mechanisms by which chromatin regulates cellular differentiation and heritability across eukaryotes.

## Figures and Tables

**Table 1:** Genes encoding known or predicted TFs and a non-DNA-binding adapter protein with roles in white-opaque switching.

**Core White-Opaque Transcriptional Regulators**					
**Gene Name**	**Orf19 #**	**Known Effect on White-Opaque Switch in Mutant Strain**			
		**White to Opaque ^1^**	**Opaque to White ^1^**	**Other Functions**	**References**
*AHR1*	Orf19.7381	1.96	0.13	Adherence	[32]
*CZF1*	Orf19.3127	0.05	0.06	Filamentation	[33]
*EFG1*	Orf19.610	24.0	<0.02	Filamentation, Metabolism	[34,35]
*SSN6* *	Orf19.6798	N/A ^OP^	<0.04	Filamentation	[36,37]
*WOR1*	Orf19.4884	>0.05	N/A ^WH^	Adherence	[38]
*WOR2*	Orf19.5992	>0.03	N/A ^WH^	Iron homeostasis	[39]
*WOR3*	Orf19.467	0.42	0.26		
*WOR4*	Orf19.6713	>0.08	N/A ^WH^		
**Auxiliary White-Opaque Transcriptional Regulators**					
**Gene Name**	**Orf19 #**	**Known Effect on White-Opaque Switch in Mutant Strain**			
		**White to Opaque ^1^**	**Opaque to White ^1^**	**Other Functions**	**References**
*AAF1*	Orf19.7436	0.88	0.38	Adherence	[40]
*AFT2*	Orf19.2272	0.36	0.59	Iron metabolism, Stress response, Adherence	[41,42]
*ARG81*	Orf19.4766	1.75	0.44	Adherence	[43]
*ASG1*	Orf19.166	0.05	0.05	Filamentation	[44]
*ASH1*	Orf19.5343	0.83	0.04	Filamentation, Metabolism	[45,46]
*BAS1*	Orf19.6874	1.49	1.15	Filamentation	[47]
*BCR1*	Orf19.723	2.21	N/A ^WH^	Adherence, Biofilm formation, Drug resistance	[43,48,49,50]
*BRG1*	Orf19.4056	1.87	0.66	Filamentation, Biofilm formation	[48,51,52]
*CAP1*	Orf19.1623	0.67	0.23	Drug resistance, Stress response, Apoptosis	[53,54,55,56,57]
*CAS5*	Orf19.4670	1.45	0.43	Drug resistance, Stress response, Cell cycle	[58,59,60]
*CPH1*	Orf19.4433	0.46	0.39	Filamentation, Mating	[61,62,63,64]
*CPH2*	Orf19.1187	0.44	0.70	Filamentation	[65]
*CRZ1*	Orf19.7359	1.86	0.18	Drug resistance, Stress response, Calcineurin pathway	[66,67,68,69,70]
*CSR1*	Orf19.3794	1.02	2.56	Zinc ion homeostasis, Filamentation	[71,72]
*CTA4*	Orf19.7374	0.88	0.17	Stress response, Drug resistance	[44,73]
*CTA7*	Orf19.4288	2.37	0.48		
*CUP2*	Orf19.5001	0.81	0.63	Stress response	[74]
*CUP9*	Orf19.6514	4.74	0.07	Filamentation	[75]
*DAL81*	Orf19.3252	0.16	0.57	Adherence	[76]
*DPB4*	Orf19.2088	0.32	0.41	Filamentation	[77]
*ECM22*	Orf19.2623	1.31	0.46		
*EFH1*	Orf19.5498	1.69	0.61	Metabolism	[34]
*FCR1*	Orf19.6817	0.52	0.63	Drug resistance	[78]
*FGR15*	Orf19.2054	0.06	4.78	Filamentation	[79]
*FLO8*	Orf19.1093	<0.04	N/A ^WH^	Filamentation, CO^2^ sensing	[80,81]
*GAL4*	Orf19.5338	<0.04	0.77	Metabolism	[82]
*GIS2*	Orf19.3182	0.86	0.11	Drug resistance	[83]
*GRF10*	Orf19.4000	1.37	0.14	Filamentation, Metabolism	[47,84]
*GZF3*	Orf19.2842	0.08	1.70	Stress response	[83]
*HAP2*	Orf19.1228	<0.04	0.62	Iron homeostasis	[39]
*HAP3*	Orf19.4647	0.34	1.30	Stress response	[39]
*HAP31*	Orf19.517	0.04	0.79	Stress response, Drug resistance	[39,83]
*HAP41*	Orf19.740	0.10	1.14	Stress response	[39]
*HAP42*	Orf19.1481	0.49	0.54		
*HAP5*	Orf19.1973	0.15	0.33	Stress response, Metabolism	[39,85]
*HCM1*	Orf19.4853	18.4	0.27	Stress response, Filamentation	[39,86]
*HFL1*	Orf19.3063	<0.05	2.11	DNA replication	[87]
*INO2*	Orf19.7539	<0.04	0.28	Transcription	[88]
*INO4*	Orf19.837.1	0.33	0.81	Transcription	[88]
*ISW2*	Orf19.7401	3.40	2.89	Stress response	[89]
*KAR4*	Orf19.3736	0.51	1.23	Mating	[90,91]
*LYS143*	Orf19.4776	7.25	0.88	Biofilm formation	[43]
*LYS144*	Orf19.5380	1.29	0.42	Biofilm formation	[43]
*MAC1*	Orf19.7068	0.05	0.63	Copper ion homeostasis, Filamentation	[92]
*MIG1*	Orf19.4318	<0.03	1.37	Metabolism	[93,94,95]
*MIG2*	Orf19.5326	1.62	0.63	Metabolism	[93]
*MSN4*	Orf19.4752	1.88	0.21	Biofilm formation	[43]
*NDT80*	Orf19.2119	0.10	1.87	Drug resistance, Biofilm formation	[48,96]
*NTO1*	Orf19.5910	2.80	0.66	Stress response	[89]
*OPI1*	Orf19.1543	4.03	0.42	Filamentation, Metabolism	[97,98]
*PTH2*	Orf19.4231	2.96	0.24		
*RAP1*	Orf19.1773	16.0	0.64	Telomere recombination, Filamentation	[99,100,101]
*RBF1*	Orf19.5558	N/A ^OP^	<0.03	Filamentation	[102]
*RCA1*	Orf19.6102	0.11	0.53	CO^2^ sensing, Drug resistance	[103,104]
*REP1*	Orf19.7521	0.68	2.43	Drug resistance	[105]
*RFG1*	Orf19.2823	1.05	2.12	Filamentation, Stress response	[106,107,108]
*RFX1*	Orf19.3865	1.78	1.67	Stress response	[109]
*RFX2*	Orf19.4590	1.46	0.58	Stress response	[109]
*RME1*	Orf19.4438	2.15	0.57	Drug resistance	[110]
*RPN4*	Orf19.1069	18.2	0.67	Intracellular proteolysis	[111,112]
*RTG1*	Orf19.4722	0.47	0.35	Metabolism	[113]
*RTG3*	Orf19.2315	0.36	0.46	Metabolism	[113]
*SEF2*	Orf19.1926	1.09	0.31		
*SFL1*	Orf19.454	0.88	1.95	Flocculation, Filamentation	[114,115]
*SKN7*	Orf19.971	1.13	0.65	Stress response	[116]
*SKO1*	Orf19.1032	0.56	0.42	Stress response, Filamentation	[117,118,119]
*STP2*	Orf19.4961	9.18	0.15	Metabolism	[120]
*STP4*	Orf19.909	3.25	0.47	Metabolism	[120]
*SWI4*	Orf19.4545	0.22	1.02	Cell cycle	[121]
*TYE7*	Orf19.4941	2.04	0.97	Metabolism	[82]
*UGA33*	Orf19.7317	0.99	0.60	Adherence	[43]
*UME6*	Orf19.1822	0.62	2.02	Filamentation, CO^2^ sensing	[122,123,124,125]
*UME7*	Orf19.2745	0.50	1.44	Adherence	[43]
*UPC2*	Orf19.391	0.94	3.13	Drug resistance, Metabolism	[126,127,128,129]
*WAR1*	Orf19.1035	0.31	0.94	Stress response	[130]
*XBP1*	Orf19.5210	0.16	0.85	Stress response	[39]
*ZCF16*	Orf19.2808	1.47	1.19	Biofilm formation	[131]
*ZCF17*	Orf19.3305	1.31	2.22	Adherence	[43]
*ZCF2*	Orf19.431	0.59	0.36	Stress response	[132,133]
*ZCF20*	Orf19.4145	0.66	0.46	Iron homeostasis	[134]
*ZCF21*	Orf19.4166	0.25	0.02		
*ZCF22*	Orf19.4251	1.77	0.93		
*ZCF24*	Orf19.4524	0.96	0.31	Stress response	[39]
*ZCF25*	Orf19.4568	8.48	0.34		
*ZCF27*	Orf19.4649	0.53	1.46	Filamentation	[43]
*ZCF30*	Orf19.5251	1.08	0.60		
*ZCF31*	Orf19.5924	0.42	3.24	Stress response	[83]
*ZCF34*	Orf19.6182	0.35	0.22	Stress response	[83]
*ZCF7*	Orf19.1685	4.73	0.37		
*ZCF8*	Orf19.1718	0.48	0.46	Adherence	[76]
*ZFU2*	Orf19.6781	0.52	2.26		
*ZFU3*	Orf19.6888	0.20	0.06	Biofilm formation	[48,131]
*ZMS1*	Orf19.5026	0.36	0.84		
	Orf19.1150	1.22	0.78		
	Orf19.1274	0.70	1.20		
	Orf19.1577	0.89	0.68		
	Orf19.1757	1.04	0.61		
	Orf19.217	0.60	0.57		
	Orf19.2476	1.91	2.49		
	Orf19.2612	2.38	1.40		
	Orf19.2961	7.02	2.05		
	Orf19.3928	5.71	0.23		
	Orf19.7098	7.77	1.10		

^1^ Fold changes in switch frequencies for each deletion mutant strain are calculated relative to an isogenic wildtype reference strain. “N/A ^OP^” indicates that the deletion mutant strain was opaque-locked and the white to opaque or opaque to white switch frequency could not be determined. “N/A ^WH^” indicates that the deletion mutant strain was white-locked, or failed to yield stable opaque colonies, and the opaque to white switch frequency could not be determined. “*” indicates a non-DNA-binding adaptor protein. Blank cells indicate that the information is unknown. Switch frequency data in this table was obtained from [9].

**Table 2:** Genes encoding known or predicted writers, erasers, chromatin remodelers and histone chaperones analyzed for their impacts on white-opaque switching.

Gene Name	Orf19#	Protein Function	Known Effect on White-Opaque Switch in Mutant Strain		Reference
			Wh --> Op ^1^	Op --> Wh ^1^	
*YNG2*	Orf19.878	Histone Acetyltransferases (Writers)	23.8	0.01	[5]
*SPT10*	Orf19.2361		no effect	no effect	[135]
*HPA2*	Orf19.6323		no effect	no effect	[135]
*RTT109*	Orf19.7491		0.10	6.98	[157]
*NAT4*	Orf19.4664		0.12	3.42	[135]
*SAS2*	Orf19.2087		no effect	no effect	[135]
*HAT1*	Orf19.779		7.60	0.13	[140]
*ELP3*	Orf19.7387		no effect	no effect	[135]
*SET1*	Orf 9.6009	Histone Methyl Transferases (Writers)	1.73	0.98 ^3^	[135]
*SET2*	Orf19.175		no effect	no effect	[135]
*DOT1*	Orf19.740		no effect	no effect	[135]
*HDA1*	Orf19.2606	Histone Deacetylases (Erasers)	2.73	1.06 ^3^	[29,31] *
*HDA2*	Orf19.6952		3.33	no data	[158]
*HDA3*	Orf19.7344		3.67	no data	[158]
*RPD3*	Orf19.2834		33.3	49.7	[137]
*RPD31*	Orf19.6801		2.85	1.23 ^3^	[136]
*HST1*	Orf19.4761		1.29 ^3^	0.37	[135]
*HST2*	Orf19.2580		0.04	1.86 ^3^	[135]
*HST3* ^4^	Orf19.1934		6.00	no effect	[24]
*HOS1*	Orf19.4411		no effect	no effect	[135]
*HOS2*	Orf19.5377		0.13	2.29	[135]
*HOS3*	Orf19.2772		no effect	no effect	[135]
*SET3*	Orf19.7221		0.16	2.71	[135]
*PHO13* ^2^	Orf19.4444	Phosphatases (Erasers)	0.93 ^3^	5.01	[135]
*ORF19.4736*	Orf19.4736		no effect	no effect	[135]
*SWR1*	Orf19.1871	Chromatin Remodelers	16.0	0.01	[5]
*CAC2*	Orf19.6670	Histone Chaperones	3.75	2.53	[139]
*HIR1*	Orf19.2099		no effect	no effect	[139]

^1^ Fold changes in switch frequencies for each deletion mutant strain are calculated relative to an isogenic wildtype reference strain. ^2^ The protein encoded by *PHO13* has been shown to lack protein phosphatase activity and is instead involved in metabolism [159,160]. ^3^ The effect on white-opaque switching is not significant. ^4^ This strain is an *HST3/hst3* hemizygous mutant strain. * The *hda1/hda1* deletion mutant strain was investigated in both referenced articles with similar findings. All switch frequencies reported in this table originate from the indicated references.

## Data Availability

Not applicable.
